# Preserved emotional awareness of pain in a patient with extensive bilateral damage to the insula, anterior cingulate, and amygdala

**DOI:** 10.1007/s00429-014-0986-3

**Published:** 2015-01-11

**Authors:** Justin S. Feinstein, Sahib S. Khalsa, Tim V. Salomons, Kenneth M. Prkachin, Laura A. Frey-Law, Jennifer E. Lee, Daniel Tranel, David Rudrauf

**Affiliations:** 1 Department of Neurology, University of Iowa, Iowa City, IA 52242 USA; 2 Department of Psychology, University of Iowa, Iowa City, IA 52242 USA; 3 Department of Psychology and School of Community Medicine, University of Tulsa, Tulsa, OK 74104 USA; 4 Laureate Institute for Brain Research, 6655 S. Yale Avenue, Tulsa, OK 74136-3326 USA; 5 Semel Institute for Neuroscience and Human Behavior, University of California, Los Angeles, Los Angeles, CA 90095 USA; 6 School of Psychology and Clinical Language Sciences, University of Reading, Reading, RG6 6AL UK; 7 Department of Psychology, University of Northern British Columbia, Prince George, BC V2N 4Z9 Canada; 8 Department of Physical Therapy and Rehabilitation Science, University of Iowa, Iowa City, IA 52242 USA; 9 Laboratory of Functional Imaging, INSERM U678s/UPMC, 75013 Paris, France

**Keywords:** Brain lesion, Consciousness, Emotion, Feeling, Limbic system

## Abstract

Functional neuroimaging investigations of pain have discovered a reliable pattern of activation within limbic regions of a putative “pain matrix” that has been theorized to reflect the affective dimension of pain. To test this theory, we evaluated the experience of pain in a rare neurological patient with extensive bilateral lesions encompassing core limbic structures of the pain matrix, including the insula, anterior cingulate, and amygdala. Despite widespread damage to these regions, the patient’s expression and experience of pain was intact, and at times excessive in nature. This finding was consistent across multiple pain measures including self-report, facial expression, vocalization, withdrawal reaction, and autonomic response. These results challenge the notion of a “pain matrix” and provide direct evidence that the insula, anterior cingulate, and amygdala are not necessary for feeling the suffering inherent to pain. The patient’s heightened degree of pain affect further suggests that these regions may be more important for the regulation of pain rather than providing the decisive substrate for pain’s conscious experience.

## Introduction

This past decade has witnessed the emergence of a new paradigm in neuroscience, where inferences about psychological states are made based on certain overlapping patterns of brain activation found during functional neuroimaging experiments. A prime example has been the invocation of the “pain matrix”, a distributed set of brain regions that exhibit a reliable and graded increase in activation in response to increasing levels of pain. This pattern of activation is evident across a large number of functional neuroimaging studies and includes regions of the periaqueductal gray, thalamus, insula, anterior cingulate cortex (ACC), and somatosensory cortices (Davis 2000; Duerden and Albanese 2013; Johnstone et al. 2012; Peyron et al. 2000; Tracey and Mantyh 2007; Wager et al. 2013). Pain, however, is not a unitary phenomenon and a dissociation between neural systems subserving pain affect and pain sensation has been proposed, with limbic regions of the matrix encoding the emotional aspects of pain and primary sensory regions encoding the location and intensity of pain sensation (Price 2000; Rainville et al. 1997). Interestingly, limbic regions typically activated during pain, especially the anterior insula and dorsal ACC, are not only active during moments of physical pain, but also during moments of social pain, such as when being ostracized from a social gathering (Eisenberger et al. 2003), vicariously experiencing the pain of another (Singer et al. 2004), feeling the heartbreak of a recent break-up (Kross et al. 2011) or the grief following the death of a loved one (O’Connor et al. 2008). The distinct overlap of neural activation patterns associated with social and physical pain has led some to conclude that the shared representations within the insula and ACC represent the critical substrate underlying the emotional experience of pain, thus providing a plausible neural explanation for a diverse set of complex social constructs ranging from why rejection hurts to how humans are capable of experiencing empathy and behaving in an altruistic manner (Eisenberger and Lieberman 2004; Eisenberger 2012; Hein et al. 2010). In essence, activity within a network of regions referred to here as the “affective pain matrix”—which features the insula and ACC, but may also include other limbic structures such as the amygdala (Neugebauer et al. 2004; Simons et al. 2014; Veinante et al. 2013)—has become commensurate with the emotional experience of pain.

Functional neuroimaging is fundamentally a correlative technique, and when conducted in isolation, it does not allow for the extrapolation of causal brain–behavior relationships. Prior to inferring that activation within limbic regions of the pain matrix is necessary for the emotional experience of pain, it is important to first conduct complementary investigations using other techniques that allow for more causal inferences. The lesion method provides a more direct test of causality and can be utilized to help constrain the interpretation of functional neuroimaging data (Feinstein 2013). In the case of pain, the lesion method allows an investigator to determine whether or not a particular brain region is “necessary” for its experience.

Beginning in the early 1950s, reports emerged of a profound akinetic mutism that was accompanied by a marked indifference to pain in patients presenting with large bilateral lesions that impacted the ACC (Nielsen and Jacobs 1951; Barris and Schuman 1953). Psychosurgeries commenced shortly thereafter, where patients with chronic intractable pain from a variety of etiologies received focal ACC lesions (Hurt and Ballantine 1973) or circumscribed anterior cingulotomies (Foltz and White 1962) in an effort to reduce their pain. Post-surgical observations highlighted a selective reduction in pain-related anxiety and distress, despite the fact that most patients continued to experience the sensation of pain (Foltz and White 1962). However, no quantitative sensory testing was ever conducted and the primary endpoint variable was a lack of opiate withdrawal symptoms to pain medication. Unfortunately, most of these studies were poorly controlled and lacked objective criteria and a proper control group for assessing the efficacy of the procedure. In a more recent study, the majority of patients receiving an anterior cingulotomy reported “mild” improvement in pain 1 year post-surgery, as well as a reduced tendency to ruminate about pain; nevertheless most patients continued to experience “significant levels of pain” at follow-up (Cohen et al. 2001). Quite strikingly, the only two studies to conduct quantitative sensory testing in a post-cingulotomy patient both found increases (rather than decreases) in pain intensity and unpleasantness ratings to painful thermal stimuli (Davis et al. 1994; Greenspan et al. 2008).

In contrast to the cingulate, very little research has examined pain in patients with bilateral amygdala damage. The main exception is the case of patient HM, who showed markedly diminished perception of heat-induced pain that may have been confounded by the presence of peripheral neuropathy (Hebben et al. 1985).

With respect to the insular cortex, a close inspection of past studies examining pain in patients with damage to this region reveals a mixed set of findings. Geschwind (1965) first speculated that lesions to the insula could disconnect the secondary somatosensory cortex from the limbic system causing a condition known as pain asymbolia, where the patient remains able to feel the sensation of pain without experiencing the associated emotional response (e.g., distress or behavioral withdrawal). Consistent with Geschwind’s hypothesis, six stroke patients with unilateral lesions to the insula were found to have pain asymbolia when tested during the acute phase after their stroke (Berthier et al. 1988). However, the lesions often extended into adjacent territories (including parietal operculum, secondary somatosensory cortex, and supramarginal gyrus) making it unclear as to whether the insula damage was the primary cause for the deficit. No studies have replicated the original finding of pain asymbolia following insula damage. Instead, a recent study found that damage to the posterior insula has a more deleterious effect on temperature perception than pain (Baier et al. 2014). And while several studies have reported increased pain thresholds following unilateral insula lesions (Greenspan et al. 1999; Schon et al. 2008), others have reported pain hypersensitivity (Starr et al. 2009), and in some cases focal damage to the posterior insula and parietal operculum can actually induce pain (Bowsher 2006; Thomas-Anterion et al. 2010; Veldhuijzen et al. 2010) and trigger a debilitating central neuropathic pain syndrome termed *operculo-insular pain* (Garcia-Larrea et al. 2010). The condition can be so agonizing that in one of the originally reported case studies the patient committed suicide in order to stop the pain (Biemond 1956). An important limitation of all the previous studies is that they were conducted in patients with unilateral insula lesions.

**Fig. 1:**
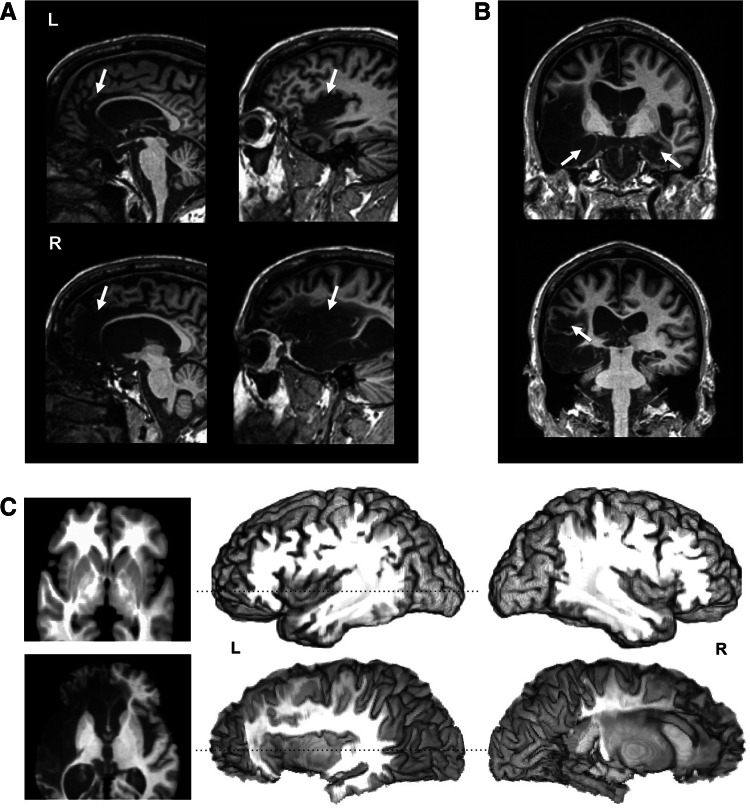
Roger’s brain. **a** Sagittal MRI slices showing bilateral lesions to the ACC (*leftmost* *images*) and insula (*rightmost images*). **b** Coronal MRI slices showing bilateral lesions to the amygdala (*top*) and right secondary somatosensory cortex (*bottom*). **c** 3D digital “dissection” of the insular cortex: *top* lateral view of the brain of a healthy non-brain damaged participant, revealing the gyrations of the insular cortex; *bottom* lateral view of Roger’s brain, highlighting the absence of an insular cortex; *left* axial MRI slices corresponding to the dashed lines on the 3D-images. All MRI slices are shown in radiological convention. Volumetric analyses (Philippi et al. 2012) reveal that his lesion encompasses 90 % of the insula, 99 % of the ACC, and 100 % of the amygdala. The lesion extends beyond these regions into other limbic territories with more extensive damage in the right hemisphere. The entire right insula is destroyed and the damage in the posterior sector extends into parietal operculum, secondary somatosensory cortex, and the underlying white matter. The vast majority of the left insula is also destroyed with the exception of a small island of tissue in the left dorsal anterior insula that appears to be functionally disconnected from the rest of the brain (Philippi et al. 2012). Although the ACC has been destroyed bilaterally, the more dorsal and posterior aspects of Brodmann area 32 appear to be spared in the left hemisphere; however, this remaining tissue is dorsal to the paracingulate sulcus, and is therefore considered part of the paracingulate cortex (and not the ACC proper). Of note, Roger’s lesion has largely spared the brainstem, thalamus, and primary and secondary somatosensory cortices. The only exception is the aforementioned damage to the right secondary somatosensory cortex, as well as some localized atrophy in the right thalamus and right pons. The reader is referred to Fig. 2 and Feinstein et al. 2010 and Philippi et al. 2012 for additional brain scans and a more detailed account of Roger’s damage

**Fig. 2:**
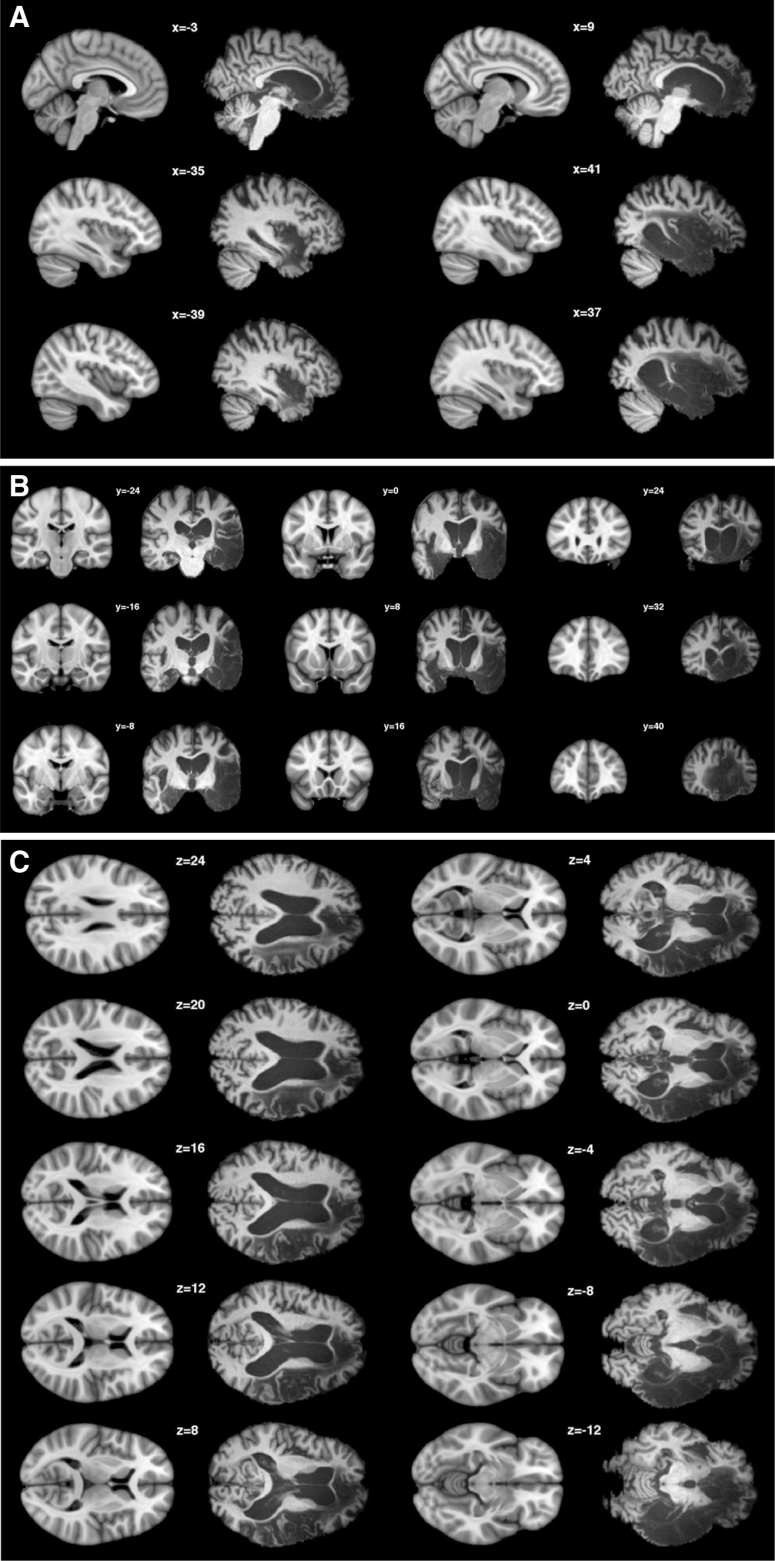
Roger’s brain in comparison to the standard MNI brain. **a** Sagittal, **b** coronal, and **c** axial MRI slices through Roger’s brain placed next to the same slice from the standard MNI brain

Based on the available lesion evidence, there have been no definitive studies to date that have addressed the necessary role of the affective pain matrix with regard to the experience of pain. The previous studies all involved patients with either unilateral insula lesions or small bilateral ACC lesions, but never a patient with damage that encompassed both structures. The ideal test case would require a lesion patient with extensive bilateral damage that not only subsumed both the insula and ACC, but also other key limbic structures implicated in pain such as the amygdala (Neugebauer et al. 2004; Simons et al. 2014; Veinante et al. 2013). Here we present such a case, an extraordinarily rare neurological patient known as Roger, whose brain damage encompasses all of the core limbic structures commonly associated with pain, bilaterally, including the insula and ACC, as well as the amygdala (Figs. 1, 2). We reasoned that if these structures are indeed necessary for the emotional experience of pain, then Roger’s damage should reduce or abolish such experience.

## Methods

The Institutional Review Board at the University of Iowa approved all study procedures and written informed consent was obtained from the patient and his family prior to conducting this study.

### Participant

Roger is a 55-year-old fully right-handed male with 16 years of education. At the age of 28, he survived a life-threatening episode of herpes simplex encephalitis (HSE), a viral attack that triggers a process of necrosis within the brain causing the “total disintegration of the affected tissue” (Hierons et al. 1978). In this case, HSE destroyed most of his limbic system, bilaterally, including his insula, ACC, and amygdala (Figs. 1, 2). Of note, the term limbic system has been used to describe Roger’s brain damage since virtually all of the key structures typically defined as comprising the limbic system have been bilaterally damaged in Roger (Feinstein et al. 2010). Detailed descriptions of his medical history and analyses of his lesion and neuropsychological profile have been reported elsewhere (Feinstein et al. 2010; Philippi et al. 2012). Remarkably, much of Roger’s cognitive abilities are within the normal range, including his intellectual functioning, speech, language, attention, working memory, and metacognition. His main presenting deficits include a dense global amnesia, along with anosmia and ageusia.

Roger’s subjective pain ratings were compared to a group of 29 healthy non-brain-damaged male subjects (average age: 29 years; range 18–55) using an identical cold pressor task and similar procedures from a previously published dataset (Lee et al. 2010).

### Imaging of Roger’s brain

Three T1-weighted magnetic resonance imaging (MRI) scans were acquired on a 3.0-Tesla Siemens Trio MRI scanner (MPRAGE, AC-PC aligned coronal acquisition; TR = 2,530 ms; TE = 3 ms; TI = 800 ms; flip angle = 10°; FOV = 256 × 256 mm^2^; slice thickness = 1 mm). The images were bias field corrected and registered together with a rigid body transformation and a sinc interpolation (AIR 3.08). The three scans were then averaged together in order to reduce motion artifacts, increase the signal-to-noise ratio, and enhance the contrast-to-noise ratio between gray and white matter (see Fig. 1). The images were then converted into MNI space (see Fig. 2).

### Stimuli and procedure

To assess the experience of pain, we utilized the cold pressor paradigm, a gold standard that has been repeatedly used over the past century to safely induce transient states of intense pain (Edes and Dallenbach 1936; Lovallo 1975; Rainville et al. 1992). Roger underwent four cold pressor immersions (two left-hand trials and two right-hand trials), each on a separate day to avoid the effects of pain summation. During each cold pressor trial, Roger’s hand was immersed (up to the wrist) in circulating water maintained at a temperature of 0 °C (32 °F). Water was circulated continuously to maintain target water temperature and avoid localized warming around the hand. The forearm was supported using a soft armrest and the hand was maintained at a constant immersion depth. Prior to each cold pressor trial, Roger underwent a baseline warm water immersion, placing the same hand in a different tank of water maintained at 33 °C (91.4 °F). The warm water trials served as a control condition and normalized limb temperature prior to each cold pressor test. For all trials, a white curtain was hung between Roger and the immersion tank, blocking his ability to see his immersed hand (in either the warm or cold water). Trials were conducted in a single-blinded fashion such that Roger was not informed beforehand whether his hand would be immersed in warm or cold water.

Throughout each immersion, Roger continuously rated his moment-to-moment level of pain (instructions detailed below) using a 10-cm electronic visual analog scale (eVAS) along both sensory and affective dimensions. Pain ratings (recorded to the nearest millimeter) were transmitted from the digital linear potentiometer to a laptop computer at a sampling rate of 50 Hz. A stopwatch was used to calculate the total immersion time. During each immersion, Roger’s facial and vocal responses were recorded using a digital video camera and we also recorded continuous measures of heart rate, skin conductance, and facial electromyography (EMG) of the corrugator.

### Self-ratings

For each hand, there were two different types of trials: “sensory” and “affective”. During the sensory trials, Roger continuously rated the intensity of his pain, ranging from “No Pain” to “Worst Pain Imaginable”. During the affective trials, Roger continuously rated how “unpleasant” or “bothersome” the pain felt to him, ranging from “Not at all unpleasant” to “Extremely unpleasant”. For all immersions, rating instructions were repeated every 30 s because of his amnesia. Prior to each immersion, the difference between pain affect and pain sensation was described to Roger using a standardized set of instructions (Price et al. 1983): “There are two aspects of pain which we are interested in measuring: the intensity, how strong the pain feels, and the unpleasantness or how disturbing the pain is for you. The distinction between these two aspects of pain is like listening to a radio. As the volume increases, I can ask you how loud it sounds or how unpleasant or bothersome it is to hear. The intensity of pain is like loudness; the unpleasantness of pain is like how disturbing or bothersome the sound is. Please indicate how intense or unpleasant/bothersome this task is when we ask you.”

### Facial coding

All facial coding was performed by an expert coder (KM Prkachin) using the Facial Action Coding System (Ekman et al. 2002). Previous work (Prkachin 1992; Prkachin and Solomon 2008) suggests that four facial actions comprise the bulk of a prototypical facial expression of pain: (1) brow-lowering; (2) tightening the eyelids or raising the cheeks (orbit tightening); (3) nose wrinkling or upper-lip raising (levator contraction); and (4) eye closure. Each of the first 3 facial actions was coded on a 6-point intensity scale (from 0 = no action, 1 = minimal action to 5 = maximal action). Eye closure was coded on a binary scale (0 = eyes open, 1 = eyes closed). A composite score was computed by summing the 4 facial action scores together (0 = no trace of a pain face to 16 = maximum expression of a pain face). Video clips of equal durations (corresponding to the first 50 s of immersion) were created for all four cold pressor and four warm water immersions. The eight video segments were randomized prior to coding and all facial coding was performed without sound so that the rater was blinded to the condition being viewed. Actions were coded on a frame-by-frame basis at a time resolution of 67 ms per frame.

### Psychophysiology

Physiological data (including heart rate, skin conductance, and corrugator facial EMG) were recorded continuously during all trials with an MP100 acquisition unit (Biopac Systems, Inc). Heart rate was collected via lead II configuration, at a sampling rate of 250 Hz. EMG responses were recorded from the corrugator muscle, at a sampling rate of 1,000 Hz. Skin conductance level was recorded using two electrodes placed on the thenar and hypothenar eminences on the non-immersed hand, at a sampling rate of 1,000 Hz. For all physiological measures, change scores were computed moment-to-moment based upon the averaged 10-s period of time immediately preceding each immersion.

## Results

### Primary pain evaluation

#### Pain withdrawal

Prior to each immersion (both warm and cold), Roger was instructed to keep his hand in the water for as long as possible. He was further instructed that he could remove his hand if the pain became intolerable. Unbeknownst to Roger, the maximum trial length was 5 min for cold pressor immersions and 3 min for warm water immersions. Roger’s hand remained in the water for the entirety of all warm water immersions. Conversely, for all four cold pressor immersions, Roger reached his tolerance threshold and withdrew his hand from the water before the maximum allocated time frame (average tolerance time: 87 s; range 37–151 s). In comparison, a group of 29 healthy male subjects had an average tolerance time that was over twice as long as Roger (average: 185 s; SD: 107 s; range 25–300 s). At all withdrawal time points, Roger reached his tolerance threshold faster than the comparison subjects. For example, at 37 s (Roger’s shortest trial), 90 % of subjects still had their hand in the water, and at 151 s (Roger’s longest trial), 59 % of subjects still had their hand in the water.

#### Self-report of pain

**Fig. 3:**
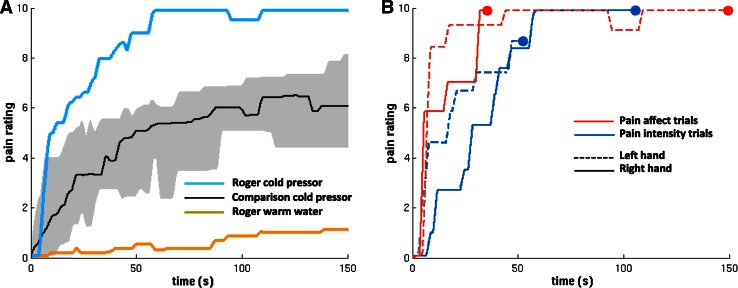
Self-ratings of pain. Roger’s real-time subjective ratings of pain using a 10-cm electronic visual analog scale. **a** Roger’s average level of pain across all four immersions for both the cold pressor and warm water trials. The *black line* represents the median cold pressor pain ratings in the healthy comparison sample and the *shaded gray* region represents the comparisons’ 25th–75th percentile. **b** Roger’s individual online ratings for each of the four cold pressor immersions. The ratings for the pain intensity trials range from “No Pain” (0) to “Worst Pain Imaginable” (10). The ratings for the pain affect trials range from “Not at all Unpleasant” (0) to “Extremely Unpleasant” (10). The *closed circles* represent the moment when Roger withdrew his hand from the water, thus terminating the trial

During all four cold pressor trials, Roger’s real-time subjective ratings of pain (both sensory and affective) indicated that he subjectively experienced extreme levels of pain that peaked within the first minute of each immersion (Fig. 3). For 3 of the trials, his subjective ratings reached the maximum level (eVAS = 10/10), and for the other trial, he withdrew his hand shortly before reaching the maximum (eVAS = 8.7/10). In contrast, his pain ratings remained at or near the floor throughout the warm water immersions (Fig. 3a). Throughout each cold pressor trial, Roger’s level of pain was elevated well beyond the 75th percentile of the average ratings from the comparison sample (Fig. 3a). There were no marked differences between his reported level of pain during right-handed immersions vs. left-handed immersions (Fig. 3b). His ratings of pain unpleasantness showed a tendency to reach their peak faster than his ratings of pain intensity (Fig. 3b).

### Secondary pain evaluation

#### Pain interview

Roger had no difficulty understanding the instructions of the cold pressor task, as clearly evident by his divergent subjective ratings during cold versus warm water trials. In order to further assess his knowledge about pain, we conducted a separate interview (transcribed below) where Roger demonstrated an astute conceptual understanding of the distinction between sensory and affective components of pain, and how the latter could be regulated based on situational factors.

Interviewer: Imagine someone lit a match and touched you with it and then I asked you two questions, how warm was it versus how painful was it?

Roger: It was too warm… it was burning hot. It hurt.

Interviewer: So how are these two questions different?

Roger: It was warm enough to burn and because it burned it was painful.

Interviewer: Can something be warm and not painful?

Roger: Yes. The fireplace across the room is a lot different than putting your arm in it.

Interviewer: Can something be painful and not warm?

Roger: Yes. Swallowing an ice cube! (Jokingly) Cool the esophagus off.

Interviewer: Okay. Let’s say you swallowed an ice cube and I asked you how painful was it and how much did it bother you?

Roger: It might be more of that [referring to the latter].

Interviewer: So what’s the difference between these two questions: how painful and how much did it bother you?

Roger: Painful would be what you remember right away and how much did it bother you might be enough to teach you not to do it again.

Interviewer: So what’s an example of something that might be painful but doesn’t bother you?

Roger: I have to poke my finger and get a little spot of blood and do a blood test. I’m diabetic. It’s painful, but I have to do it.

Interviewer: So because you have to do it, it doesn’t bother you as much?

Roger: Yep.

Interviewer: What would you say Roger is the difference between emotional pain and physical pain?

Roger: Emotional pain you can not find a spot (pointing to the body) and put a bandage on it.

#### Facial expression of pain

**Fig. 4:**
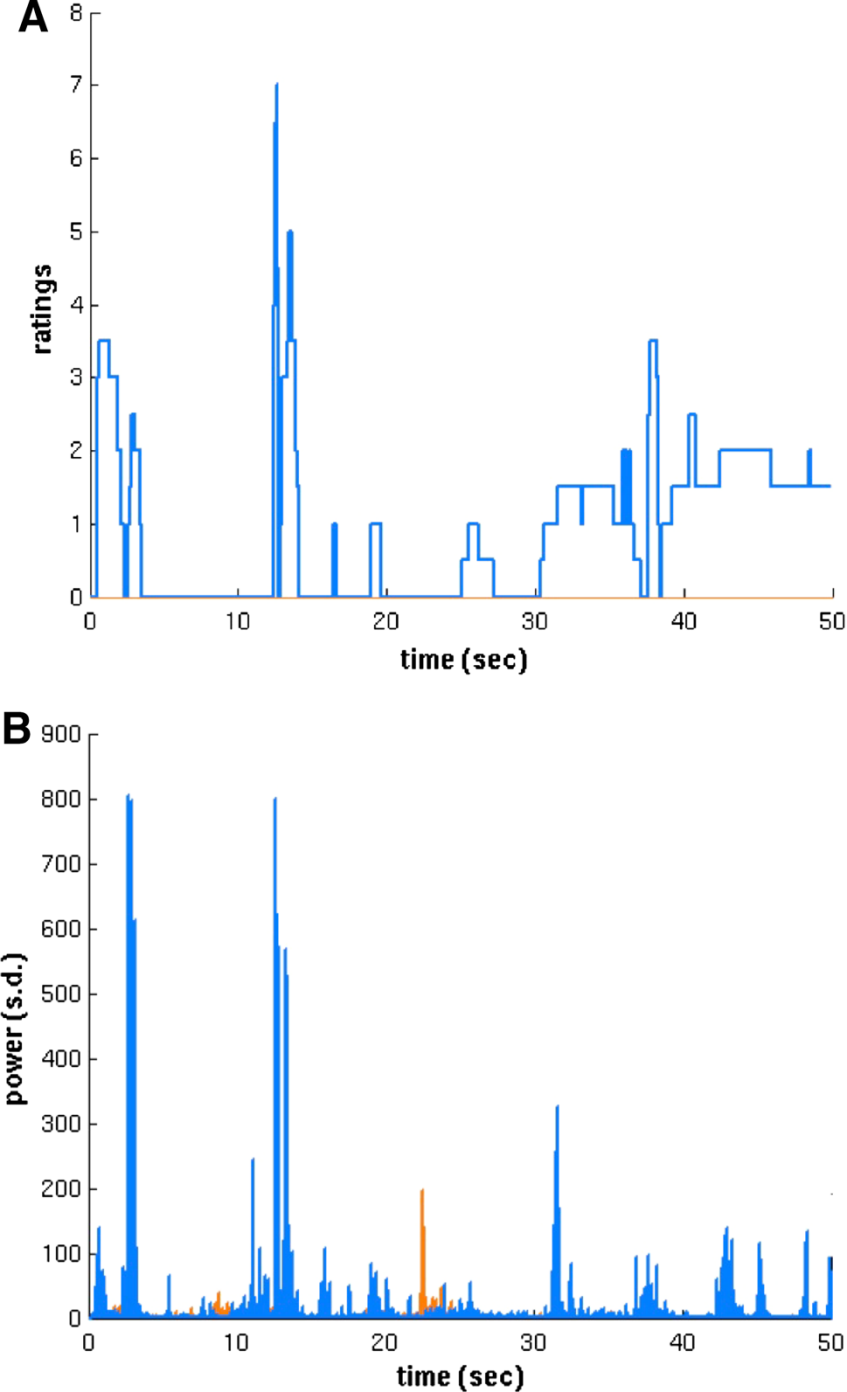
Pain facial expressions. **a** Roger’s average pain face composite score during cold pressor trials (*blue* *line*) and warm water trials (*orange line*). **b** Average corrugator EMG responses (expressed in standard deviations of the power, with respect to baseline) during cold pressor trials (*blue line*) and warm water trials (*orange line*)

During the cold pressor immersions, Roger’s face was coded as having at least some degree of pain 35.7 % of the time (Fig. 4a), with essentially no differences between the left and right hand immersions (35.4 vs. 36.1 %). During the time periods when Roger’s face was coded as expressing pain, his average pain face composite score across immersions was 2.52 (SD = 1.54), with the left-hand immersions evoking more intense facial expressions than right hand immersions [average composite score for left hand vs. right hand: 3.29 (SD = 1.35) vs. 1.64 (SD = 1.26)]. All of these average composite scores fall within the normal range of scores obtained in healthy male participants undergoing a similar cold pressor procedure (average composite score of 2.45 (SD = 3.15); scores computed using data from Prkachin 1992). In contrast to the cold pressor trials, Roger’s face displayed no signs of pain during the warm water trials (i.e., pain was coded 0 % of the time). Roger’s corrugator EMG responses, a well-studied physiological index of distress and unpleasantness (Lang et al. 1993), were considerably higher during the cold pressor immersions versus warm water immersions (Fig. 4b). As expected, the time points of his peak corrugator EMG responses often corresponded to the time points where his facial expression of pain was coded as most intense.

#### Pain vocalization

Roger exhibited intense pain vocalizations throughout each cold pressor immersion, whereas such vocalizations were absent during the warm water immersions. These vocalizations were exaggerated in terms of frequency, volume intensity, and prosodic inflection with respect to those typically exhibited by healthy comparison participants. Although subjects will occasionally make spontaneous comments about their painful experience, Roger’s commentary was disinhibited and explosive in nature (Table 1).

**Table 1:** Roger’s pain vocalizations

Day #1—left hand
Warm water (total time = 3:00)
Time 1:06 → It’s not unpleasant at all
Time 1:40 → No pain
Cold water (total time = 2:31)
Time 0:00 → Wa-hoo! Wow! Yes, that is cold! Wow
Time 0:14 → Wow. Ya. Eww
Time 0:34 → Wa-ew! Ew-how!
Time 0:44 → Extremely unpleasant
Time 1:02 → It feels extremely unpleasant
Time 1:42 → Oww
Day #2—right hand
Warm water (total time = 3:00)
Time 0:07 → Comfortable. Comfortable, no pain
Time 0:37 → No pain
Time 1:00 → None
Cold water (total time = 1:47)
Time 0:03 → No ice cubes, but it’s ice water!
Time 0:08 → Oww
Time 0:14 → Ew-how
Time 0:27 → We-ye. Ha
Time 0:31 → A lot stronger, more intense signals being sent than the hot water. Wow
Time 0:50 → Wow-ha. Ow! It is pretty bad. Wow. Ya
Time 1:04 → Ew-ow
Time 1:32 → It is that bad [pointing to rating scale]. Worst
Day #3—left hand
Warm water (total time = 3:00)
Time 0:12 → It feels good. Hurts so good (making joke). Warm water
Cold water (total time = 0:54)
Time 0:01 → Wy-ha! Ew-ha! Woah, ahh, eww
Time 0:14 → Ay!
Time 0:18 → Wow. Whew-hew-hew
Time 0:36 → Oww. Ew, oww, eww!
Time 0:43 → Ow! Ew
Time 0:48 → It is bad! Owww! Ow. I want it out!
Day #4—right hand
Warm water (total time = 3:00)
Time 0:48 → Not the hottest or the coldest.
Cold water (total time = 0:37)
Time 0:01 → Ay-ya-ya! Woah
Time 0:15 → Wow. Who. Ow-we-ha!
Time 0:17 → It feels cold all the way through
Time 0:22 → Wow! Ow! Wow. Ow. Wow-ew. Wow! Ow!
Time 0:32 → That is bad! It is bad. Wow! Wow. Oww! Ow

Roger’s pain-related vocalizations transcribed for each immersion. The spelling attempts to characterize the literal enunciation for each of his vocalizations. Exclamation marks indicate vocalizations that were emitted with high levels of intensity. Time represents the amount of time (in minutes and seconds) that his hand had been immersed in the water

#### Autonomic response

**Fig. 5:**
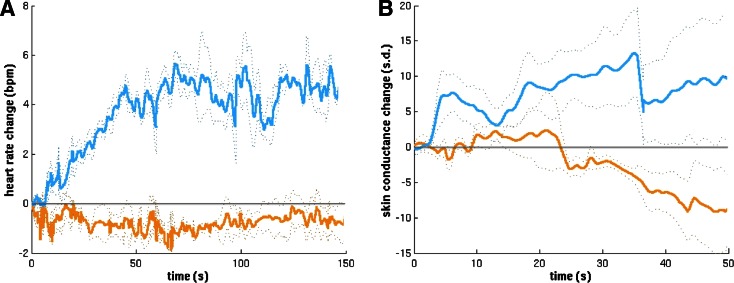
Heart rate and skin conductance changes. Roger’s average autonomic response during cold pressor trials (*blue line)* and warm water trials (*orange line*). **a** Mean change from baseline in heart rate (beats per minute). **b** Mean skin conductance level (standard deviations from baseline). The *gray dotted lines* correspond to ±1SD across trials. The dip in average skin conductance around 37 s corresponds to the end of one of the trials when Roger withdrew his hand

During warm water immersions, Roger’s average heart rate and skin conductance tended to either remain the same or slowly decrease over time with respect to baseline (Fig. 5). In contrast, during cold pressor immersions Roger’s heart rate (Fig. 5a) gradually increased over the course of the first minute and remained elevated for the remainder of the trial, and his skin conductance (Fig. 5b) showed a rapid increase during the first 10 s, followed by a brief dip, and then a slow ramping for the remainder of the trial.

## Discussion

This study provided the unique opportunity to examine pain in a rare encephalitis patient with extensive bilateral damage to core limbic structures commonly associated with pain, including the insula, ACC, and amygdala. Against expectations, the patient’s expression and experience of pain was found to be intact across multiple pain measures including self-report, facial expression, vocalization, withdrawal reaction, and autonomic response. This finding was consistent across four separate cold pressor immersions, testing both the left and right hand, with no notable differences between hands, and no notable differences between the patient’s report of pain affect versus pain sensation. Moreover, the patient’s experience of pain was discriminative in nature, being present during cold water immersions and absent during warm water immersions, despite being blinded to the testing condition. The patient’s preserved experience of pain in the face of bilateral lesions encompassing the insula, ACC, and amygdala, demonstrates that these structures are not necessary for consciously experiencing the suffering inherent to pain.

### Theoretical implications

These findings have important theoretical implications for the notion of a “pain matrix”. Previous work has already raised the question about whether the pain matrix is specific to nociception, and the evidence shows that activation within the matrix is not sufficient for the experience of pain and can be induced by a variety of non-nociceptive stimuli (Iannetti and Mouraux 2010; Mouraux et al. 2011). Here, we extend this body of work by showing that key structures of the pain matrix are also not necessary for the experience of pain, including pain’s affective dimension (Price 2000; Rainville et al. 1997). In this context, it is important to note that Roger’s damage also includes structures posited to play a more basic role in pain sensation, including the posterior insula (bilaterally) and the adjacent parietal operculum and secondary somatosensory cortex (in the right hemisphere). Based on this additional damage and the noted presence of pain following stimulation of both sides of the body, it can be further deduced that these lesioned sensory structures are also not necessary for the experience of pain, calling into question recent claims that the dorsal posterior insula is the brain’s “primary cortex for pain” (Garcia-Larrea 2012). Thus, the core limbic structures of the pain matrix, including insula and ACC, are neither necessary nor sufficient for the experience of pain.

Such a conclusion refutes the reverse inferences being made in functional neuroimaging investigations of social pain. For example, activation of the ACC during studies of social rejection may not be the causal factor explaining why “rejection hurts” (Eisenberger et al. 2003; Eisenberger and Lieberman 2004; Eisenberger 2012). Likewise, activation of the anterior insula during studies of empathy may not represent the vicarious experience of another’s pain (Singer et al. 2004). The results in Roger raise the possibility that the overlapping activation found in the insula and ACC during states of physical and social pain does not necessarily reflect the experience of pain itself. Indeed, patients with congenital insensitivity to pain showed normal levels of insula and ACC activity when observing pain in others, even though they themselves are unable to feel pain (Danziger et al. 2009). On the other hand, psychopaths showed abnormally high levels of insula activity when observing pain in others, leading the authors of this study to conclude, “the role of the insula in emotion and empathy is complex and far from being understood” (Decety et al. 2013). Collectively, these data challenge the core assumptions underlying the neural connection between physical and social pain (*cf.* Iannetti et al. 2013), while underscoring the importance of resisting causal attributions based purely on functional neuroimaging data (Feinstein 2013).

Beyond pain, the insula and ACC are the two most commonly activated structures in any functional neuroimaging investigation of emotion and feeling (Craig 2009; Phan et al. 2002). While it would be tempting to conclude that the insula and ACC are the brain’s primary substrate for emotional experience, the case of Roger reveals that neither region is actually necessary for such experience to occur. This is an important point since several investigators have recently claimed that the anterior insula is the necessary substrate underlying all forms of emotional awareness (Craig 2009; Gu et al. 2013). We have previously shown that Roger’s self-awareness is remarkably preserved across a large battery of tests (Philippi et al. 2012), including a measure of interoceptive awareness (Khalsa et al. 2009). In this study, we demonstrate that many aspects of Roger’s emotional awareness are also preserved, a finding which casts grave doubt on the assertion that the insula is the brain’s most important substrate for feeling (Craig 2015; Damasio 2003).

### Hyperpathic pain

Quite unexpectedly, not only did Roger feel pain, but his pain experience was at times excessive in nature and potentially hyperpathic. The International Association for the Study of Pain characterizes hyperpathic pain as “an abnormally painful reaction to a stimulus” that “is often explosive in character” (*cf.* http://www.iasp-pain.org). Consistent with this definition, Roger’s subjective rating of pain during cold pressor trials was often at the maximum level, well above the 75th percentile of the corresponding ratings from the comparison group (Fig. 3a). Likewise, he reached his pain tolerance threshold and withdrew his hand much earlier than the majority of subjects in the comparison group. Perhaps the clearest evidence of hyperpathic pain was in his vocalizations, which were explosive and disinhibited (Table 1). These indications of hyperpathic pain in Roger will need to be further investigated using more precise thermal and mechanical pain-induction techniques, and compared to an age- and race-matched sample. Despite these limitations, Roger’s data suggest that the limbic structures commonly associated with pain may play a fundamental role in pain regulation. Under this view, the missing regions in Roger’s brain would impair his ability to control and downregulate his pain responses. This would be in line with a large body of literature on the role of the ACC in adaptive control, not only for pain, but also cognition and autonomic arousal (Shackman et al. 2011; Botvinick et al. 2004; Critchley et al. 2003). Such an interpretation is also consistent with the significant disturbances found in circuitry involving the insula, ACC, and amygdala in patients and animals under conditions of chronic pain (Borsook and Becerra 2006; Bushnell et al. 2013; Veinante et al. 2013). However, Roger is not a chronic pain patient and it is unclear how his brain damage might reflect alterations in the chronic pain state. Together, these findings suggest that the functional role of the limbic structures comprising the pain matrix may be more aligned with the adaptive regulation and response to pain rather than providing the decisive substrate for pain’s conscious experience, affective or otherwise.

### How does Roger feel pain?

The question remains as to which brain regions might be supporting Roger’s preserved experience of pain. We attempted to investigate this question with Roger using fMRI. Over the course of many different runs (collected over multiple days), Roger was unable to refrain from pain-related movements, an unfortunate byproduct of his aforementioned hyperpathic responses. In spite of all our efforts at correcting his movement artifacts, the pain fMRI data were not exploitable. We hope that methodological solutions will be found for rendering future pain-related fMRI studies with Roger possible. Until then, we can only speculate as to which brain regions might be supporting his pain experience. From a neuroanatomical perspective, nearly all pain signals traverse through nuclei within the brainstem and thalamus, both of which are largely intact in Roger and could be playing a critical role in his pain experience. While Roger’s ACC is destroyed bilaterally, there is some remaining tissue in the left hemisphere that corresponds to a dorsal region of Brodmann area 32 within the paracingulate gyrus. This region partially overlaps with a recent functional neuroimaging meta-analysis of pain-related activations (*cf.* Box 1 and Fig. 2 in Shackman et al. 2011) suggesting that adjacent territories lying dorsal to the anterior midcingulate cortex (including the paracingulate gyrus and supplementary motor area) may be just as important for pain as the ACC itself. Such an interpretation is consistent with the observation that most patients with akinetic mutism—a state that is often accompanied by a complete indifference to pain—have large bilateral lesions that typically impact both the ACC and the supplementary motor area (Damasio and Van Hoesen 1983). Roger’s secondary somatosensory cortex in the left hemisphere is also intact and might provide a viable compensatory route for the damage to this region in his right hemisphere. Of note, activation in the secondary somatosensory cortices appears to have one of the strongest relationships with the “subjective reality of pain” (Raij et al. 2005). Another possibility to consider is the primary somatosensory cortices, which are intact in both hemispheres, and have previously been shown to play a vital role in Roger’s preserved interoceptive awareness for cardiovascular sensations (Khalsa et al. 2009). Interestingly, a recent fMRI study tested two lesion patients with unilateral left insula damage and found “dramatically elevated” levels of activation in the primary somatosensory cortex related to the patients’ higher pain ratings during noxious heat stimulation (Starr et al. 2009). Another noteworthy point of the Starr et al. (2009) study was the surprising absence of activation in both patients’ right insular cortex during painful stimulation of the right leg. This provides further evidence that the insula is not essential for the experience of pain. Nevertheless, it is worth considering the possibility that the small island of tissue remaining in Roger’s left dorsal anterior insula (accounting for less than 10 % of total insular volume) could be contributing to his pain experience. Several points argue against this possibility: (1) Patient 2 in the Starr et al. (2009) study had damage that completely subsumed the left anterior insula, yet he continued to experience pain; (2) there are anecdotal reports of pain being experienced by another encephalitic patient with 100 % complete bilateral insula destruction (Damasio et al. 2013); and (3) in a previous study, we found that the tissue in Roger’s left anterior insula was both structurally and functionally disconnected from the rest of his brain (Philippi et al. 2012). Based on this evidence, it is highly unlikely that this small island of tissue would be playing a prominent role in Roger’s preserved pain experience.

The case of Roger establishes that the emotional experience of pain can be instantiated by brain structures outside of those traditionally presumed to be critical for pain affect, thus highlighting the widely distributed nature of pain processing in the brain (Coghill et al. 1999). Due to the chronicity of Roger’s damage, substantial recovery of function is plausible via reorganization and transfer to other brain systems (Rudrauf 2014). It is important to emphasize that in such a scenario, the very possibility of recovery would imply that the damaged regions are not necessary for such experience to occur (in contrast, for instance, to early visual cortices which are necessary for visual awareness). This brings forth an educated guess about Roger’s case, namely that his intact affective experience of pain is due to plasticity, which helped preserve a vital function for survival by maintaining his affective response to pain despite damage to regions that might normally serve this function. In other words, the adaptive role of pain affect is so essential that the brain may automatically rewire in service of self-preservation. Consequently, the neural circuitry underlying pain and the associated feelings of suffering and distress is more complicated than previously thought, with multiple pathways and built-in redundancy allowing for maximal adaptation and resilience in the face of brain injury.
